# Exacerbated LPS/GalN-Induced Liver Injury in the Stress-Sensitive Wistar Kyoto Rat Is Associated with Changes in the Endocannabinoid System

**DOI:** 10.3390/molecules25173834

**Published:** 2020-08-23

**Authors:** Marykate Killilea, Daniel M. Kerr, Beth M. Mallard, Michelle Roche, Antony M. Wheatley

**Affiliations:** 1 Physiology, School of Medicine, National University of Ireland Galway, H91W5P7 Galway, Ireland; m.killilea2@gmail.com (M.K.); danny.kerr@nuigalway.ie (D.M.K.); B.Mallard@massey.ac.nz (B.M.M.); 2 Pharmacology and Therapeutics, School of Medicine, National University of Ireland Galway, H91W5P7 Galway, Ireland; 3 Galway Neuroscience Centre, National University of Ireland Galway, H91W5P7 Galway, Ireland

**Keywords:** stress, cannabinoid, liver injury, WKY, inflammation

## Abstract

Acute liver injury (ALI) is a highly destructive and potentially life-threatening condition, exacerbated by physical and psychological stress. The endocannabinoid system plays a key role in modulating stress and hepatic function. The aim of this study was to examine the development of acute liver injury in the genetically susceptible stress-sensitive Wistar-Kyoto (WKY) rat compared with normo-stress-sensitive Sprague Dawley (SD) rats, and associated changes in the endocannabinoid system. Administration of the hepatotoxin lipopolysaccharide/D-Galactosamine (LPS/GalN) resulted in marked liver injury in WKY, but not SD rats, with increased alanine aminotransferase (ALT), aspartate aminotransferase (AST) and glutamate dehydrogenase (GLDH) plasma levels, significant histopathological changes, increased hepatic pro-inflammatory cytokine expression and caspase-3 activity and expression and reduced Glutathione (GSH) activity. Furthermore, compared to SD controls, WKY rats display increased anandamide and 2-Arachidonoylglycerol levels concurrent with decreased expression of their metabolic enzymes and a decrease in cannabinoid (CB)_1_ receptor expression following LPS/GalN. CB_1_ antagonism with AM6545 or CB_2_ agonism with JWH133 did not alter LPS/GalN-induced liver injury in SD or WKY rats. These findings demonstrate exacerbation of acute liver injury induced by LPS/GalN in a stress-sensitive rat strain, with effects associated with alterations in the hepatic endocannabinoid system. Further studies are required to determine if the endocannabinoid system mediates or modulates the exacerbation of liver injury in this stress-sensitive rat strain.

## 1. Introduction

Acute liver injury (ALI) is a rare, highly destructive and potentially life-threatening condition. Characterised by sudden onset of severe hepatic dysfunction, ALI has been associated with viral hepatitis, drug overdose, exposure to toxins and unknown causes [1,2]. The severity of injury can vary but may lead ultimately to acute liver failure and it is associated with hepatic encephalopathy [3] and multisystem organ failure [4]. Acute liver failure as a result of ALI is associated with a 30–80% mortality rate depending on the etiology and currently there is no treatment except transplantation of the organ [5]. Several animal models have been developed in order to uncover the pathophysiological processes that underlie ALI. One such model involves administration of D-galactosamine (GalN), a specific inhibitor of hepatocyte transcription, in combination with the bacterial endotoxin lipopolysaccharide (LPS) [6], which results in the pathological features of ALI similar to what is seen clinically [7]. ALI in this model has been shown to be mediated by the release of pro-inflammatory mediators including tumour necrosis factor alpha (TNF-α), interleukins (IL)-6 and IL-1β produced by Kupffer cells following the binding of LPS to Toll-like receptor 4 (TLR4) [8]. Release of these immune mediators results in the infiltration of inflammatory cells into the liver and the induction of apoptotic liver injury [9].

Psychological and physical stress is well known to alter physiological function and has been shown to increase the risk of developing, and exacerbate already existing, liver disease (for review see [10]). Meta-analysis has revealed a significant association between high levels of anxiety/psychological stress and liver disease mortality in patients [11]. However, the biological mechanisms underpinning how stress impacts ALI development and progression have not been investigated. The Wistar-Kyoto (WKY) rat is a genetically stress-sensitive strain of rat that has been used to study the impact of genetic susceptibility to stress on physiological function and as a model of stress-related disorders such as depression, anxiety and stress-induced hyperalgesia [12,13,14,15]. Research including studies on cholesterol regulation in the liver following bile duct ligation [16] and the effect of the autonomic nervous system on CCl_4_-induced liver disease [2] have been conducted in the WKY rat. However, no study to date has investigated if liver function, or changes that occur in response to liver injury, is altered in these stress-sensitive rats when compared to normo-stress-sensitive comparators.

The endocannabinoid system modulates a host of physiological functions including liver function and the stress response. Composed of the G-protein coupled receptors, cannabinoid receptor 1 and 2 (CB_1_ and CB_2_), endogenous cannabinoid ligands including *N*-arachidonoylethanolamine (anandamide, AEA) and 2-arachidonoylglycerol (2-AG) and the enzymes responsible for their synthesis and degradation; this system is expressed throughout cells and tissues in the body. In the liver, endocannabinoids, notably AEA and 2-AG, have also been identified as regulators of hepatic haemodynamics [17], lipid metabolism and fibrogenesis (for review see [18,19,20,21]). Furthermore, the endocannabinoid system is an important modulator of immune function and TLR4-mediated inflammation [22]. Endocannabinoid levels in the liver are low under normal physiological conditions and have been shown to be significantly elevated following liver injury [23]. AEA has been shown to be released by Kupffer cells and lymphocytes while 2-AG is released by hepatocytes and hepatic stellate cells depending on the nature of the injury [19]. Furthermore, activation of CB_1_ receptors in the liver contributes to hepatocyte death and fibrogenesis, [24], while activation of CB_2_ receptors has been shown to have anti-inflammatory/anti-fibrogenic properties and to reverse paracetamol induced liver injury [25], cirrhosis [26,27], non-alcoholic fatty liver disease [28] and alcoholic liver disease [29] in experimental models. As such, it has been proposed that targeting this system could ameliorate the injury itself and/or reduce complications associated with these life-threatening hepatic diseases.

Thus, the aim of this study was to investigate the development of ALI in stress-sensitive WKY rats compared to normo-stress-sensitive Sprague Dawley (SD) counterparts and associated changes in the hepatic endocannabinoid system.

## 2. Results

### 2.1. WKY Rats Display Anxiety-Related Phenotype in the Open Field and Elevated Plus Maze

**Figure 1:**
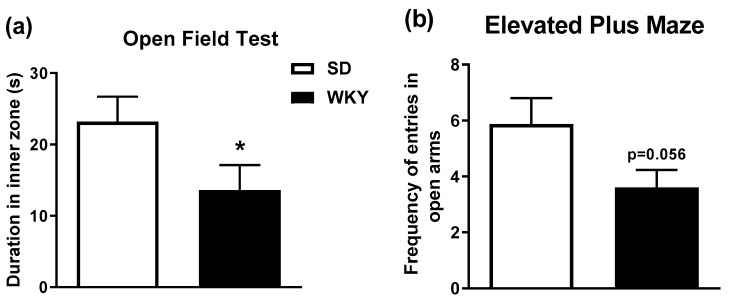
WKY rats display reduced (**a**) duration of time spent in inner zone in open field (* *p* < 0.05 vs. SD) and (**b**) frequency to enter the open arms of the elevated plus maze (*p* = 0.056). Data expressed as mean ± SEM (*n* = 15–16 per group).

WKY rats have consistently been reported to exhibit a stress-related behavioural phenotype and this was confirmed in this study where WKY rats exhibited anxiety-like behaviour in the open field and elevated plus maze. T-test analysis revealed that WKY rats exhibit a significant reduction in time spent in the inner zone of the open field test (t_30_ = 1.93 *p* < 0.05) and a decreased frequency of entry into the open arms of the elevated plus maze, although this just failed to reach statistical significance (t_30_ = 1.99 *p* = 0.056), compared to SD rats (Figure 1).

### 2.2. Histopathological Changes and Elevated Biochemical Markers of Liver Injury in WKY Rats in Response to LPS/GalN Compared to SD Counterparts

**Figure 2:**
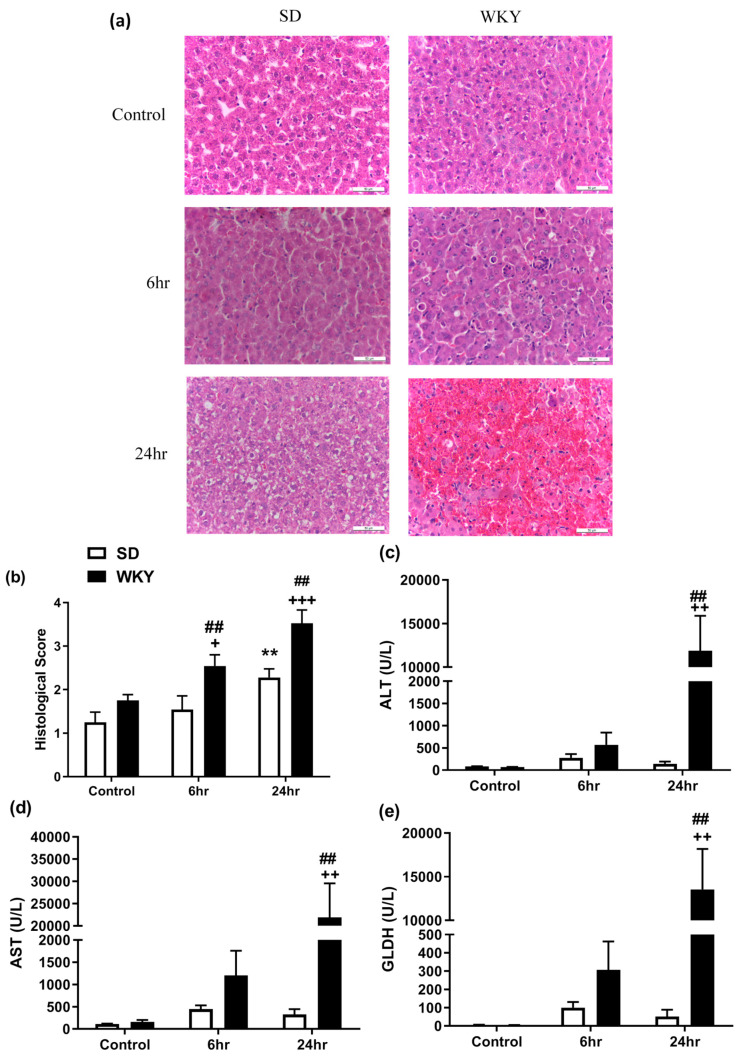
(**a**) Histological examination of liver from SD and WKY rats following saline (control) or LPS/GalN administration (6 and 24 h post administration) (H&E staining 400×. Bar = 50 µm). Effect of LPS/GalN on (**b**) hepatic histopathology score and plasma levels of (**c**) ALT (**d**) AST and (**e**) GLDH. Data expressed as mean ± SEM (*n* = 6–8). * *p* < 0.05; ** *p* < 0.01 vs. SD saline; ++ *p* < 0.01 +++ *p* < 0.001 vs. WKY saline; ## *p* < 0.01 vs. SD counterpart.

Microscopic evaluation of liver sections following LPS/GalN revealed marked structural hepatic changes identified as infiltration of immune cells into the sinusoids at 6 h and congestion, degradation of hepatocellular architecture, haemorrhage and extensive apoptosis and necrosis at 24 h, in both SD and WKY rats (Figure 2a; Supplementary Figure S2). Analysis of histopathological scoring revealed an effect of rat strain (F_1,39_ = 19.54 *p* < 0.001) and time (F_2,39_ = 14.79 *p* < 0.001). Post hoc analysis revealed that WKY rats displayed significant liver injury at 6 and 24 h post LPS/GalN compared to both saline control and SD counterparts (Figure 2b). The histological data were accompanied by a significant effect of strain (F_1,40_ = 21.18 *p* < 0.001), time (F_2,40_ = 8.08 *p* = 0.001) and strain x time interaction (F_2,40_ = 9.16 *p* < 0.001) on liver:body weight ratio. Post hoc analysis revealed that WKY, but not SD, rats displayed a reduced liver:body weight ratio 24 h post LPS/GalN when compared to SD counterparts (SD: 3.63 ± 0.14 vs. WKY: 2.72 ± 0.16, *p* < 0.01). In line with the histological findings, analysis revealed a significant effect of rat strain, time and strain x time interaction on alanine aminotransferase (ALT) (strain F_1,36_ = 8.77 *p* = 0.005; time F_2,36_ = 8.38 *p* = 0.001; interaction F_2,39_ = 8.50 *p* < 0.001), aspartate aminotransferase (AST) (strain F_1,36_ = 8.43 *p* = 0.006; time F_2,36_ = 7.91 *p* = 0.001; interaction F_2,39_ = 7.85 *p* = 0.001) and glutamate dehydrogenase (GLDH) (strain F_1,36_ = 8.87 *p* = 0.006; time F_2,36_ = 8.52 *p* < 0.001; interaction F_2,39_ = 8.53 *p* < 0.001) plasma levels. Post hoc analysis revealed that LPS/GalN administration significantly increased ALT, AST and GLDH plasma levels in the WKY rat strain 24 h post administration when compared to saline control (*p* < 0.01) and SD counterparts (*p* < 0.01), confirming the exacerbation of ALI in this rat strain. Toll-like receptor 4 (TLR4) is the primary receptor to which LPS binds to and in association with its adaptor protein MD2, results in initiation of the inflammatory responses. As such, basal expression of TLR4 and MD2 in the liver was examined in WKY vs. SD rats revealing no significant difference between the two strains (TLR4: SD: 100 ± 8.2% WKY: 99 ± 4.0, *p* > 0.05; MD2: SD: 100 ± 7.8 WKY: 99 ± 4.8, *p* > 0.05).

### 2.3. Altered Hepatic Inflammatory Profile Following LPS/GalN in WKY Compared with SD Rats

Inflammatory cytokines such as TNF-α are key mediators of LPS/GalN-induced liver injury [30] and activation of caspase-3 has been identified as a vital step in the induction of non-parenchymal cell apoptosis induced by LPS/GalN [31]. Analysis revealed an effect of strain (F_1,39_ = 4.53 *p* = 0.040) and time (F_2,39_ = 4.19 *p* = 0.023) on TNF-α mRNA expression and an effect of strain (F_1,36_ = 6.76 *p* = 0.013), time (F_2,36_ = 6.87 *p* = 0.003) and strain x time interaction (F_2,36_ = 4.73 *p* = 0.015) on IL-6 mRNA expression.

**Figure 3:**
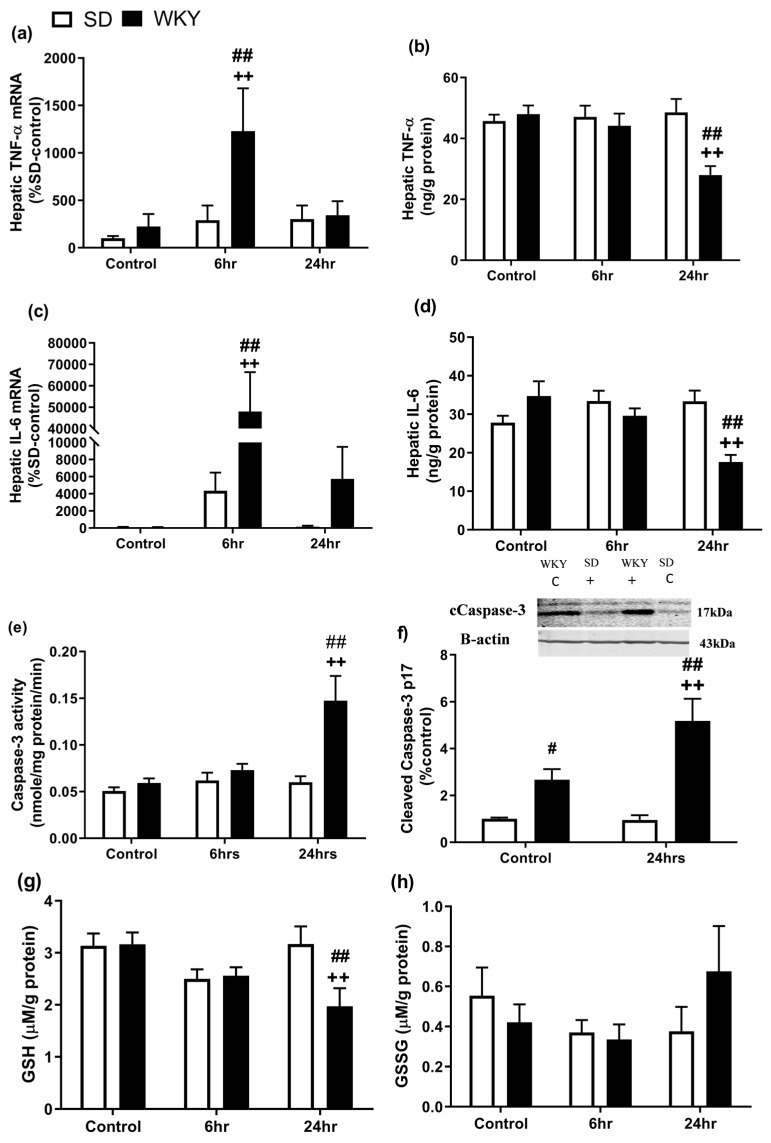
Effect of LPS/GalN on hepatic (**a**,**b**) TNF- α mRNA and protein levels, (**c**,**d**) IL-6 mRNA and protein levels, (**e**,**f**) caspase-3 activity and expression, (**g**) GSH and (**h**) GSSG activity 6 and 24 h post administration (*n* = 6–8). ++ *p* < 0.01 vs. WKY control; # *p* < 0.05, ## *p* < 0.01 vs. SD counterparts. Data expressed as mean ± SEM (*n* = 4–8).

Post hoc analysis revealed that TNFα and IL-6 mRNA levels were significantly increased in liver, 6 h post administration, in WKY rats compared to saline control (*p* < 0.01) and SD counterparts (*p* < 0.01) (Figure 3a–d). Although IL-6 expression returned to baseline levels at 24 h post LPS/GalN in SD rats, expression remained significantly elevated in WKY rats at this time point (Figure 3b) (*p* < 0.01). A two-way ANOVA revealed a significant effect of strain (F_1,34_ = 4.55 *p* = 0.04) and strain x time interaction (F_2,34_ = 4.77 *p* = 0.015) on hepatic levels of TNF-α, and time (F_2,34_ = 3.98 *p* = 0.028) and strain x time interaction (F_2,34_ = 8.58 *p* = 0.001) on hepatic IL-6 levels. Post hoc analysis revealed that both TNF and IL-6 protein levels were significantly decreased in WKY rats 24 h post administration of LPS/GalN when compared to SD counterparts (Figure 3c,d) (*p* < 0.01 vs. SD 24 h).

A two-way ANOVA revealed a significant effect of strain (F_1,42_ = 12.84 *p* < 0.001), time (F_2,42_ = 8.64 *p* < 0.001) and strain x time interaction (F_2,42_ = 6.73 *p* = 0.003) on caspase-3 activity and a significant effect of strain (F_1,20_ = 30.66 *p* < 0.001), time (F_2,20_ = 5.32 *p* = 0.032) and strain x time interaction (F_2,20_ = 5.78 *p* = 0.026) on cleaved caspase-3 expression (17 kDa). Post hoc analysis revealed that WKY rats displayed a significant increase in caspase-3 activity (Figure 3e) and cleaved caspase-3 protein expression (Figure 3f) 24 h post LPS/GalN in WKY rats when compared to saline control (*p* < 0.01) and SD counterparts (*p* < 0.01). In addition, WKY control rats exhibited an increase in cleaved caspase-3 expression when compared to SD control counterparts. There was no effect of strain or time on expression of 19 kDa caspase-3 protein.

Glutathione (GSH) depletion has been associated with increased hepatocyte susceptibility to oxidative stress [32]. Two-way ANOVA revealed a significant effect of time (F_2,41_ = 3.76 *p* = 0.032) and strain x time interaction (F_2,41_ = 3.83 *p* = 0.030) on GSH levels. Post hoc analysis revealed that hepatic levels of reduced GSH did not significantly differ between SD and WKY rats at baseline, however, these were significantly decreased in WKY rats 24 h post LPS/GalN administration, when compared to SD counterparts (*p* < 0.01) and WKY controls (*p* < 0.01) (Figure 3g). In comparison, oxidised levels of glutathione (GSSG) were not significantly different between SD and WKY rat nor altered by LPS/GalN at either 6 or 24 h post administration (Figure 3h).

### 2.4. Alterations in the Hepatic Endocannabinoid System Prior to and Following LPS/GalN in WKY and SD Rats

**Figure 4:**
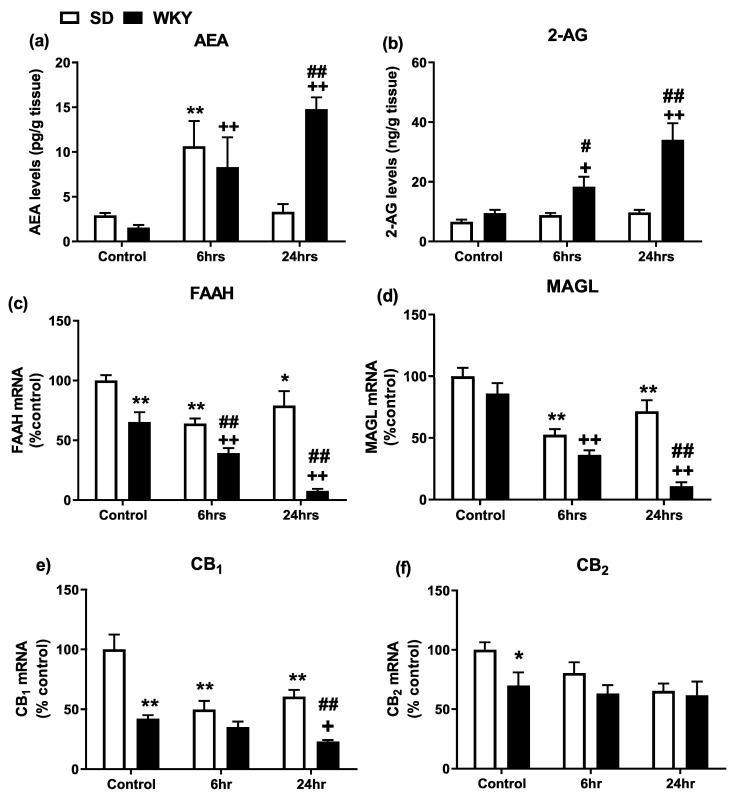
Effect of LPS/GalN on hepatic endocannabinoid system in SD and WKY rats. (**a**) AEA and (**b**) 2-AG levels. (**c**) FAAH and (**d**) MAGL mRNA expression. (**e**) CB_1_ and (**f**) CB_2_ receptor mRNA expression. * *p* < 0.05 ** *p* < 0.01 vs. SD saline control; + *p* < 0.05 ++ *p* < 0.01 vs. WKY saline control; # *p* < 0.05 ## *p* < 0.01 vs. SD counterparts. Data expressed as mean ± SEM (*n* = 6–8).

Endocannabinoid levels, the mRNA expression of enzymes and receptors for the endocannabinoid system and protein levels of CB_1_ and CB_2_ in the liver of SD and WKY rats were examined. Two-way ANOVA revealed a significant effect of time (F_2,38_ = 7.66 *p* = 0.002) and strain x time interaction (F_2,38_ = 6.90 *p* = 0.003) on hepatic AEA levels and a significant effect of strain (F_1,42_ = 29.55 *p* < 0.001), time (F_2,42_ = 12.81 *p* < 0.001) and strain x time interaction (F_2,42_ = 7.92 *p* = 0.001) on hepatic 2-AG levels. Post hoc analysis revealed that AEA levels were significantly increased 6 h post LPS/GalN administration in both SD and WKY rats compared to saline controls (Figure 4a). Although AEA levels returned to baseline levels 24 h following LPS/GalN administration in SD rats, levels remained elevated in the liver of WKY rats at the same time point. Levels of 2-AG were significantly elevated in WKY rats 6 and 24 h post LPS/GalN administration when compared to WKY control and SD counterparts (Figure 4b).

AEA is primarily metabolised by the enzyme fatty acid amid hydrolase (FAAH) while 2-AG is primarily broken down by the enzyme monoacylglycerol (MAGL). Two-way ANOVA revealed a significant effect of strain (F_1,40_ = 72.17 *p* < 0.001), time (F_2,40_ = 21.35 *p* < 0.001) and strain x time interaction (F_2,40_ = 7.66 *p* = 0.002) on hepatic FAAH expression and a significant effect of strain (F_1,40_ = 35.25 *p* < 0.001), time (F_2,40_ = 40.87 *p* < 0.001) and strain x time interaction (F_2,40_ = 9.01 *p* = 0.0006) on hepatic MAGL expression. Post hoc analysis revealed that WKY rats exhibit reduced hepatic FAAH mRNA expression when compared to SD counterparts (*p* < 0.01) (Figure 4c). LPS/GalN decreased FAAH and MAGL expression at both 6 and 24 h post administration in both SD and WKY rats (Figure 4c,d: SD 6 h vs. SD control), an effect potentiated in WKY rats (*p* < 0.01) (Figure 4c,d).

Two-way ANOVA revealed a significant effect of strain (F_1,38_ = 45.27 *p* < 0.001), time (F_2,38_ = 12.33 *p* < 0.001) and strain x time interaction (F_2,38_ = 5.43 *p* = 0.008) on hepatic CB_1_ receptor mRNA expression and a significant effect of strain (F_1,38_ = 5.17 *p* = 0.029) on hepatic CB_2_ receptor mRNA expression. Post hoc analysis revealed that the mRNA expression of CB_1_ and CB_2_ receptors was significantly reduced in WKY vs. SD rats (Figure 4e–f). CB_1_ receptor mRNA was significantly decreased at 6 and 24 h post LPS/GalN administration in SD rats, and at 24 h post administration in WKY rats, compared to corresponding saline-treated controls (Figure 4e) (*p* < 0.01).

### 2.5. Neither the Peripheral Restricted CB_1_ Receptor Antagonist AM6545 nor CB2 Receptor Agonist JWH-133 Altered LPS/GalN-Induced Liver Injury in SD or WKY Rats

**Figure 5:**
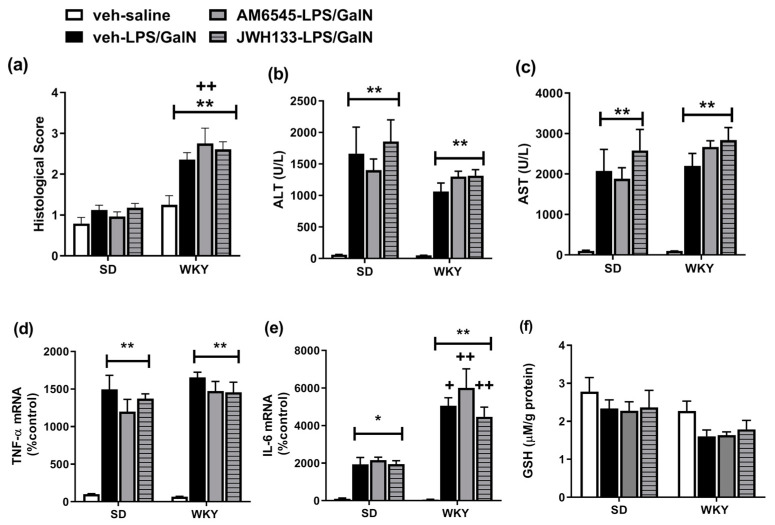
Effect of AM6545 and JWH133 on (**a**) histopathological score, (**b**) ALT (**c**) AST, (**d**) TNF-α, (**e**) IL-6 mRNA and (**f**) hepatic GSH levels in SD and WKY rats. Data expressed as mean ± SEM (*n* = 6–8). * *p* < 0.05 ** *p* < 0.01 vs. vehicle–saline counterpart + *p* < 0.05 ++ *p* < 0.01 vs. SD counterpart.

Given the changes in the hepatic endocannabinoid system associated with LPS/GalN-induced liver injury, and data demonstrating beneficial effects of CB1 antagonism and CB2 receptor agonism in several models of liver disease [20,27], the effects of modulating these receptors on ALI in SD and WKY rats was examined. Two-way ANOVA revealed a significant effect of strain (F_1,46_ = 75.47 *p* < 0.001), treatment (F_3,46_ = 7.36 *p* < 0.001) and strain × treatment interaction (F_3,46_ = 3.56 *p* = 0.021) on the histological score. Post hoc analysis revealed that LPS/GalN results in hepatic injury in WKY, but not SD rats, an effect not altered by prior pre-treatment with AM6545 or JWH133. Two-way ANOVA revealed a significant effect of strain (F_1,42_ = 5.16 *p* = 0.028) and treatment (F_3,42_ = 23.76 *p* < 0.001), on plasma ALT levels and a significant effect of treatment on plasma AST levels (Figure 5). Post hoc analysis revealed that LPS/GalN resulted in increased plasma levels of ALT and AST in SD and WKY rats, an effect not altered by pre-treatment with AM6545 or JWH133. Analysis of hepatic TNF-α revealed a significant effect of treatment (TNF-α F_3,44_ = 59.32 *p* < 0.001; IL-6 F_3,43_ = 23.42 *p* < 0.001) with expression increased following LPS/GalN treatment in SD and WKY rats, an effect not altered by prior administration of either AM6545 or JWH133. Two-way ANOVA revealed a significant effect of strain (F_1,43_ = 44.55 *p* < 0.001), treatment (F_3,43_ = 23.42 *p* < 0.001) and strain x treatment interaction (F_3,43_ = 5.24 *p* = 0.036) on IL-6 mRNA expression. Post hoc comparisons revealed that LPS/GalN significantly increased IL-6 mRNA expression in SD and WKY rats, an effect augmented in WKY rats. A significant effect of strain (F_1,48_ = 9.92 *p* = 0.002) was revealed for GSH; however, no significant differences were found between the groups following post hoc analysis.

## 3. Discussion

The results of the present study demonstrate that the stress-sensitive WKY rat exhibits an exacerbated liver injury in response to LPS/GalN, an effect accompanied by alterations in hepatic immune profiles and the endocannabinoid system. Specifically, LPS/GalN administered to WKY rats results in marked histological changes as early as 6 h post injection and enhanced hepatic apoptosis by 24 h, accompanied by elevations in plasma transaminases (ALT and AST), GLDH, hepatic pro-inflammatory cytokines and caspase-3 activity and expression, as well as reduced GSH, when compared to SD counterparts. Thus, the exacerbated liver injury following LPS/GalN in WKY vs. SD rats is likely mediated by an enhanced hepatic pro-inflammatory response and activation of apoptotic cascades. Given that the endocannabinoid system is known to modulate inflammatory pathways and hepatic function, exacerbation of LPS/GalN-induced liver injury in WKY rats may be accompanied by altered hepatic endocannabinoid function. Accordingly, the data herein demonstrate that WKY rats exhibit alteration in the endocannabinoid system when compared to SD rats—both under basal conditions and in response to LPS/GalN. However, acute systemic administration of CB_1_ antagonist or CB_2_ agonist did not alter acute liver injury or inflammation induced by LPS/GalN in SD or WKY rats. Taken together, the data indicate that WKY rats exhibit exacerbated ALI in response to LPS/GalN, an effect associated with changes in the hepatic endocannabinoid system. Further studies are required to determine if an altered hepatic endocannabinoid system may mediate or modulate the inflammatory response and subsequent exacerbated liver injury observed in this stress-sensitive strain of rat following LPS/GalN.

To our knowledge, this is the first study to examine the impact of a stress-related phenotype on the development of ALI. Our data herein demonstrated that WKY rats exhibit a greater number of infiltrating immune cells at 6 h and higher plasma levels of ALT, AST and GLDH and major hepatic damage at 24 h post LPS/GalN administration compared to SD counterparts. Although the expression of TLR4 and MD2 did not differ between SD and WKY rats, the data indicate a robust and enhanced immune response to LPS/GalN in WKY rats. LPS/GalN-induced injury has been shown to be mediated by TNF-α binding to the TNF-α receptor 1, which ultimately leads to hepatocyte apoptosis [9,30]. The current study demonstrated that LPS/GalN hepatic increases in TNF-α and IL-6 mRNA expression in WKY, but not SD, rats. Although protein levels of these cytokines were not altered, TNF-α signalling is activated approximately 60–90 min following LPS [33] and thus it is likely that LPS/GalN resulted in increases in cytokine protein levels at time points earlier than 6 h. The increase in hepatic proinflammatory mediators in turn leads to enhanced caspase-3 activity and expression, apoptosis and exacerbated liver injury in WKY rats compared with SD counterparts.

The endocannabinoid system plays a key role in mediation and the modulation of innate immune and hepatic function [20,21,27,34]. WKY rats exhibit alteration in key components of the endocannabinoid system within discrete brain regions [35,36,37] and cannabidiol has been demonstrated to attenuate depressive-like behaviour in this rat strain [38,39]. Our data confirm the stress-related phenotype of the WKY rats (anxiety-related behaviour) and extend the current literature demonstrating an altered hepatic endocannabinoid system both at baseline and in response to LPS/GalN, when compared to SD rats. Specifically, the expression of the AEA metabolising enzyme, FAAH, and expression of CB_1_ and CB_2_ receptors are all reduced in the liver of WKY vs. SD rats. Thus, WKY rats exhibit alterations in the hepatic endocannabinoid system which may predispose these animals to enhanced inflammatory responses to LPS/GalN and subsequent exacerbated ALI. Furthermore, in response to LPS/GalN, SD and WKY rats exhibit differential changes in components of the endocannabinoid system. Both AEA and 2-AG levels are enhanced in the liver of WKY rats at both 6 and 24 h following LPS/GalN, while AEA levels were only increased in SD rats 6 h post administration. LPS/GalN reduced expression of FAAH, MAGL and CB_1_ receptors in both SD and WKY rats, an effect potentiated in WKY rats. The robust decrease in expression of the metabolising enzymes, FAAH and MAGL, following LPS/GalN administration in WKY rats may be responsible for the enhanced levels of the corresponding AEA and 2-AG, respectively. Several studies have demonstrated that immune activation results in enhanced release of endocannabinoids, an effect believed to act to modulate and control the inflammatory process [34]. Accordingly, the inhibition of MAGL and associated increases in 2-AG has been shown to attenuate LPS/GalN-induced liver injury [40]. However, endocannabinoids have been shown to also induce apoptosis in hepatic stellate cells and hepatocytes depleted of GSH [41,42]. Thus, it is possible that increased endocannabinoid levels in WKY rats following LPS/GalN, and associated alterations in the activity of this system (FAAH, MAGL and CB_1_ receptors), are such that the endocannabinoid system is unable to attenuate the associated inflammatory state and instead results in endocannabinoid-induced apoptosis and exacerbated liver injury in WKY rats. Alternatively, the changes in endocannabinoid levels and expression at 24 h may be a consequence of the exaggerated immune response, apoptosis and hepatocyte destruction in WKY rats.

A wealth of data has indicated that CB_1_ receptor activation is a key mediator in the exacerbation of several liver pathologies and CB_2_ receptor activation results in anti-inflammatory and antifibrogenic effects in several models [20,25,27]. In an attempt to further elucidate the role of CB_1_ and CB_2_ receptors in ALI in the current model, the effect of acute systemic administration of CB_1_ antagonist and CB_2_ agonist on the development of hepatic inflammation and ALI in SD and WKY rats was examined. The data presented here confirm the exacerbated histopathological damage and IL-6 expression in WKY following LPS/GalN administration and demonstrate that pre-treatment with a CB_1_ antagonist or CB_2_ agonist does not alter LPS/GalN-induced liver injury in SD or WKY rats at 6 h. While these data indicate a lack of CB receptor involvement in the development of LPS/GalN-induced ALI in SD or WKY rats, it is possible that effects may be observed with higher concentrations of modulators, at a different time point (e.g., 24 h), following chronic administration or via multiple and/or non-CB_1_/CB_2_ receptor mediated mechanisms. Further studies are required in order to fully uncover the role of the hepatic endocannabinoid system in ALI, and its exacerbation in this stress-sensitive strain of rat.

Overall, the present study demonstrates that ALI is exacerbated in a stress-sensitive phenotype of rat, likely due to enhanced inflammatory responses and apoptotic processes. WKY rats exhibit dysregulation of the hepatic endocannabinoid system both at baseline and in response to LPS/GalN-induced ALI. Although, pharmacological inhibition of CB_1_ receptors or activation of CB_2_ receptors was unable to modulate LPS/GalN-induced liver injury, further studies are required in order to elucidate the possible involvement of the endocannabinoid system in ALI in high-stress-sensitive rats. Overall, these data confirm the adverse effects of a genetic susceptibility to stress on the development of ALI and associated changes in the endocannabinoid system. Such studies have important implications for understanding the interaction between stress and liver disease and identifying novel therapeutic targets for such conditions.

## 4. Materials and Methods

### 4.1. Animals

Male SD and WKY rats weighing 200 ± 10 g on arrival (Envigo Laboratories, Blackthorn, UK) were housed in groups of 3–4 per cage and allowed to acclimatise for 7 days prior to experimentation. The rats had free access to food (standard chow diet 14% protein, Envigo Laboratories, Blackthorn, UK) and water and were kept under a standard 12:12 light cycle (07:00–19:00) at a constant temperature (21 ± 2 °C). All procedures were conducted in accordance with the guidelines of the Animal Care Research Ethics Committee, National University of Ireland Galway, under license from the Health Products Regulatory Authority of Ireland in compliance with the European Communities Council directive 2010/63/EU.

### 4.2. Experimental Design

#### 4.2.1. Assessment of LPS/GalN-Induced Inflammation, Liver Injury and Changes in Endocannabinoid System in SD and WKY Rats

Prior to the induction of ALI, the stress-related phenotype of WKY rats was confirmed by exposing SD and WKY rats to the open field followed by the elevated plus maze and behaviour was recorded. Rats were habituated to handling and received intraperitoneal (i.p.) injection of 0.89% NaCl sterile saline one day prior to systemic administration of LPS/GalN (20 μg/kg Lipopolysaccharide (*Escherichia coli*, serotype 0111:B4; Sigma-Aldrich, Dublin, Ireland, Ltd.) and 200 mg/kg d-galactosamine hydrochloride (Sigma-Aldrich, Dublin, Ireland, Ltd.) dissolved in 0.89% sterile saline) or saline vehicle (*n* = 8/group). Rats were returned to their home cage and sacrificed 6 and 24 h post injection. The dose and time of administration of LPS/GalN was chosen based on an in-house pilot study and published data [43] which demonstrate that this combination of doses induces liver injury with minimal mortality. Blood samples were taken by cardiac puncture into heparinised tubes and plasma separated and stored at −80 °C, until liver enzyme analysis. Livers were excised quickly, weighed and sections were snap frozen and stored at −80 °C or fixed for histological analysis.

#### 4.2.2. The Effect of Systemic Administration of the Peripherally Restricted CB_1_ Antagonist AM6545 or CB_2_ Agonist JWH133 on LPS/GalN-Induced Inflammation and Liver Injury in SD and WKY Rats

Male SD and WKY rats were randomly assigned to one of four treatment groups: Vehicle-saline (*n* = 6), Vehicle-LPS/GalN (*n* = 8), AM6545-LPS/GalN (*n* = 8) and JWH133-LPS/GalN (*n* = 8). JWH-133 (2.5 mg/kg, Tocris, Bristol, UK), AM6545 (10 mg/kg, Tocris, Bristol UK) or Vehicle (4% DMSO: 1% Tween-80: 95% saline) were administered subcutaneously in an injection volume of 2 mL/kg followed 30 min later by a single i.p. injection of LPS/GalN (20 µg/kg LPS and 200 mg/kg GalN) or sterile saline (0.89% NaCl) administered in an injection volume of 2 mL/kg. The dose and route of AM6545 administration were chosen based on studies demonstrating an improved fatty liver and lipid profile following chronic administration and ability of acute treatment to block WIN55,212-induced decreases in core body temperature and anandamide-induced gastro-intestinal motility [44] effects devoid of central mood changes [45]. The dose of JWH133 was chosen based on studies demonstrating its anti-inflammatory effects [46,47] and attenuation of liver injury [29,48]. Blood samples were taken by cardiac puncture as described above and livers were excised quickly, weighed and sections were snap frozen and stored at −80 °C or fixed for histological analysis.

### 4.3. Anxiety-Related Behaviour

To confirm the stress-related/anxiety-like behavioural phenotype of WKY rats, SD and WKY rats were exposed to the open field test and elevated plus maze test as previously described [12]. In brief, rats were placed into the centre of a brightly illuminated circular open field arena (75 cm in diameter) and allowed to explore the novel environment for 5 min. Immediately following testing, animals were placed on an elevated plus maze for a further 5 min. Behaviour was recorded and rated with the aid of EthoVision XT11.5 video tracking software (Noldus, Wageningen, The Netherlands).

### 4.4. Liver Histology

Segments of liver were fixed in 10% neutral buffered formalin for 24 h, embedded in paraffin wax and subsequently sectioned on a microtome into 5 μm sections. Sections were then stained with haematoxylin and eosin (H&E) for histopathological analysis under a light microscope (Leica DM500, Leica Microsystems, Wetzlar, Germany). A semi-quantitative grading system was used to identify the progression of the injury based on a modified version of Suzuki’s criteria on a scale of 0–4 [49], whereby 0 indicates no discernible injury; 1 slight immune cell infiltration; 2 hepatocyte ballooning/degeneration; 3 loss of hepatic structure, increased apoptotic bodies/massive immune cell infiltration; 4 massive sinusoidal congestion with loss of hepatic structure (see Supplementary Figure S1). The grading of the slides was conducted by a researcher blinded to group identity of the samples. Three observations were scored per slide and an average of the histopathological score obtained.

### 4.5. Plasma Biochemical Analysis

Plasma levels of alanine aminotransferase (ALT), aspartate aminotransferase (AST) and glutamate dehydrogenase (GLDH) were determined using an automatic biochemical analyser (Randox Imola, RX4900, Co. Antrim, UK) in accordance with manufacturer instructions. The limit of detection for both ALT and AST were 600 U/L and was 73 U/L for GLDH.

### 4.6. Gene Expression Analysis Using qRT-PCR

RNA was extracted from liver tissue using NucleoSpin RNA II total RNA isolation kit (Macherey-Nagel, Dueren, Germany) and reverse transcribed into cDNA using a high-capacity cDNA archive kit (Applied Biosystems, Thermofisher, Cheshire, UK) as previously described [50]. The genes of interest were quantified using TaqMan gene expression assays (Applied Biosystems, UK) which contain a FAM-labelled MGB and also forward and reverse primers. RT-PCR was performed using an AB7500 instrument (Applied Biosystems, UK). The cycling conditions were 50 °C for 2 min, 95 °C for 10 min and 40 cycles of 95 °C for 15 min followed by 60 °C for 1 min. Gene IDs for the TaqMan assays were TNF-α (Rn99999017_m1), IL-6 (Rn0056142_m1), CB_1_ (Rn02758689_s1), CB_2_ (Rn03993699_s1), FAAH (Rn00577086_m1) and MAGL (Rn00593297_m1) and β-actin was used as an endogenous control to normalise gene expression data; all samples were run in duplicate. Relative gene expression was calculated using the ∆∆CT method.

### 4.7. Enzyme-Linked Immunosorbent Assay (ELISA) for TNF-α and IL-6

Liver (approximately 30 mg) was homogenised in lysis buffer (137 mM NaCl, 20 mM Tris-HCl (pH 8.0), 1% NP40, 10% glycerol, 1 mM PMSF, 10 μg/mL aprotinin, 1 μg/mL leupeptin, 0.5 mM sodium vanadate, Sigma-Aldrich, Dublin, Ireland) and centrifuged at 13,000× *g* at 4 °C for 15 min. Protein concentration was determined in supernatant using the Bradford assay. TNF-α and IL-6 were evaluated using specific commercially available ELISA kits according to supplier’s protocol (PeproTech, NJ, USA). Absorbance was read at 450 nm and cytokine levels in liver tissue are expressed as ng/g protein.

### 4.8. Caspase-3 Activity Assay

Liver was homogenised in lysis buffer; pH 7.2, containing 50 mM HEPES-KOH, 5 mM EGTA, 2 mM MgCl_2_, 10 mM KCl, 1 mM dithiothreitol and 0.1% CHAPS (Sigma-Aldrich, Dublin, Ireland). Tissue homogenates were centrifuged at 14,000× *g* at 4 °C for 15 min, supernatant was removed, and protein concentration was calculated using a Bradford protein assay. The caspase-3 activity of the supernatant was determined colorimetrically by incubating with N-Acetyl-Asp-Glu-Val-Asp-p-nitroanilide (DEVD-pNA) substrate for caspase-3 for 150 min at 37 °C. Absorbance was measured at 405 nM as caspase-3 activity and expressed as nmole/mg protein/min.

### 4.9. Hepatic Glutathione Levels

Glutathione (GSH) and oxidised glutathione (GSSG) levels were assessed in liver according to a modified protocol of the Hissan and Hilf method with use of the *ο*-pthalaldehyde (OPA) fluorescent reagent [51]. Protein concentration was determined using a Braford assay. Fluorescence was measured at 355 nm/460 nm and GSH and GSSG expressed as nmole/mg protein.

### 4.10. Western Immunoblotting

Western immunoblotting was carried out as previously described [36]. In brief, liver tissue was homogenised in RIPA lysis buffer and protein concentration determined using the Bradford assay. Then, 30µg of protein was loaded onto 12% SDS-polyacrylamide gels and transferred onto nitrocellulose membranes. Membranes were blocked in 5% milk, 0.1% Tween-20 in TBS for 1 h at room temperature and subsequently incubated with primary antibody (cleaved caspase-3: 1:1000, Cell Signalling Technologies, Massachusetts, USA) overnight and following washes incubated with fluorescent secondary antibody (1:10,000 dilution: LI-COR; Biosciences, Dublin, Ireland) for 1 h. Expression was analysed using the fluorescence ODESSEY Clx scanner (Biosciences, Dublin, Ireland). Membranes were re-probed for β-actin, which acted as the endogenous control. Densitometric analysis was carried out using Image Studio Lite software V5.2 (LI-COR Biosciences, Ireland) on bands at 17 and 19 kDa for caspase-3 expression and data normalised to the corresponding β-actin (endogenous control) (see Supplementary Figure S3 for full blot).

### 4.11. Endocannabinoid Auantification Using Liquid Chromatography-Tandem Mass Spectrometry (LC-MS/MS)

Quantitation of endocannabinoids (AEA and 2-AG) in liver tissue was carried out as previously described [50]. Liver tissue samples were sonicated in 100% acetonitrile containing deuterated internal standards (0.014 nmol AEA-d8, 0.48 nmol 2-AG-d8). Lyophilised samples and standards were evaporated, reconstituted and suspended in 65% acetonitrile. Reconstituted samples were then separated by reversed-phase gradient elution HPLC coupled to a triple quadrupole 6460 mass spectrometer (Agilent Technologies Ltd., Cork, Ireland) using electrospray-positive ionisation and multiple reaction monitoring (MRM) mode. Analytes were further quantified using Masshunter Quantitative Analysis Software (Aligent Technologies, Cork, Ireland) by radiometric analysis and results expressed as nmol or pmol per gram of tissue. The limits of detection for analyte quantifications were as follows; 1.3 pmol·g^−1^ and 12.1 pmol·g^−1^ for AEA and 2-AG, respectively.

### 4.12. Statistical Analysis

All data are presented as means ± standard error of the mean (SEM). All statistical analyses were carried out using the SPSS statistical package (IBM SPSS Statistics v21 for Microsoft Windows; SPSS Inc., Chicago, IL, USA). Normality and homogeneity of variance were assessed using the Shapiro–Wilk and Levene test. Data were analysed using an unpaired T-test or two-way analysis of variance (ANOVA) followed by the LSD post-hoc test, where appropriate. The significance level was set at *p* < 0.05. Graphical illustration of the data was constructed using GraphPad prism software for Windows version 8 (GraphPad Software, La Jolla, CA, USA).

## Supplementary Materials

The following are available online, Supplementary Figure S1: Representative images of each histopathological score. Supplementary Figure S2 Histological examination of liver from SD and WKY rats following saline (control) or LPS/GalN administration (6 and 24 hours post administration). Supplementary Figure S3: Representative full western immunoblot image of β-actin and Caspase-3.

## Abbreviations

2-AG	2-Arachidonoylglycerol
AEA	Anandamide
ALI	Acute liver injury
ALT	Alanine aminotransferase
AST	Aspartate aminotransferase
CB1	Cannabinoid receptor 1
CB2	Cannabinoid receptor 2
FAAH	Fatty acid amide hydrolase
GalN	d-Galactosamine
GLDH	Glutamate dehydrogenase
GSH	Glutathione
GSSG	Oxidised glutathione
IL	Interleukin
LPS	Lipopolysaccharide
MAGL	Monoacylglycerol lipase
SD	Sprague Dawley
MD2	Myeloid adaptor protein 2
TLR4	Toll-like receptor 4
WKY	Wistar-Kyoto
