# Supplementary material for: Exacerbated LPS/GalN-Induced Liver Injury in the Stress-Sensitive Wistar Kyoto Rat Is Associated with Changes in the Endocannabinoid System
Source: Molecules. 2020 Aug 23;25(17):3834. doi: 10.3390/molecules25173834 (PMC7504576; doi:10.3390/molecules25173834)
Supplement: Supplementary file 1 [file molecules-25-03834-s001.pdf]

Supplementary Data

Histopathological analysis was then performed by examining the stained tissue sections under light microscopy (Leica DM500, Leica Microsystems, Wetzlar, Germany). A semi-quantitative grading system was used to identify the progression of the injury based on a modified version of Suzuki's criteria on a scale of 0-4 (Chen *et al.*, 2012) whereby 0 indicates no discernible injury; 1 slight immune cell infiltration; 2 hepatocyte ballooning/degeneration; 3 loss of hepatic structure, increased apoptotic bodies/ massive immune cell infiltration and 4 massive sinusoidal congestion with loss of hepatic structure (see Suppl Fig 1). The grading of the slides were blinded to group identity of the samples and 3 observations were scored per slide and an average of the histopathological score obtained.

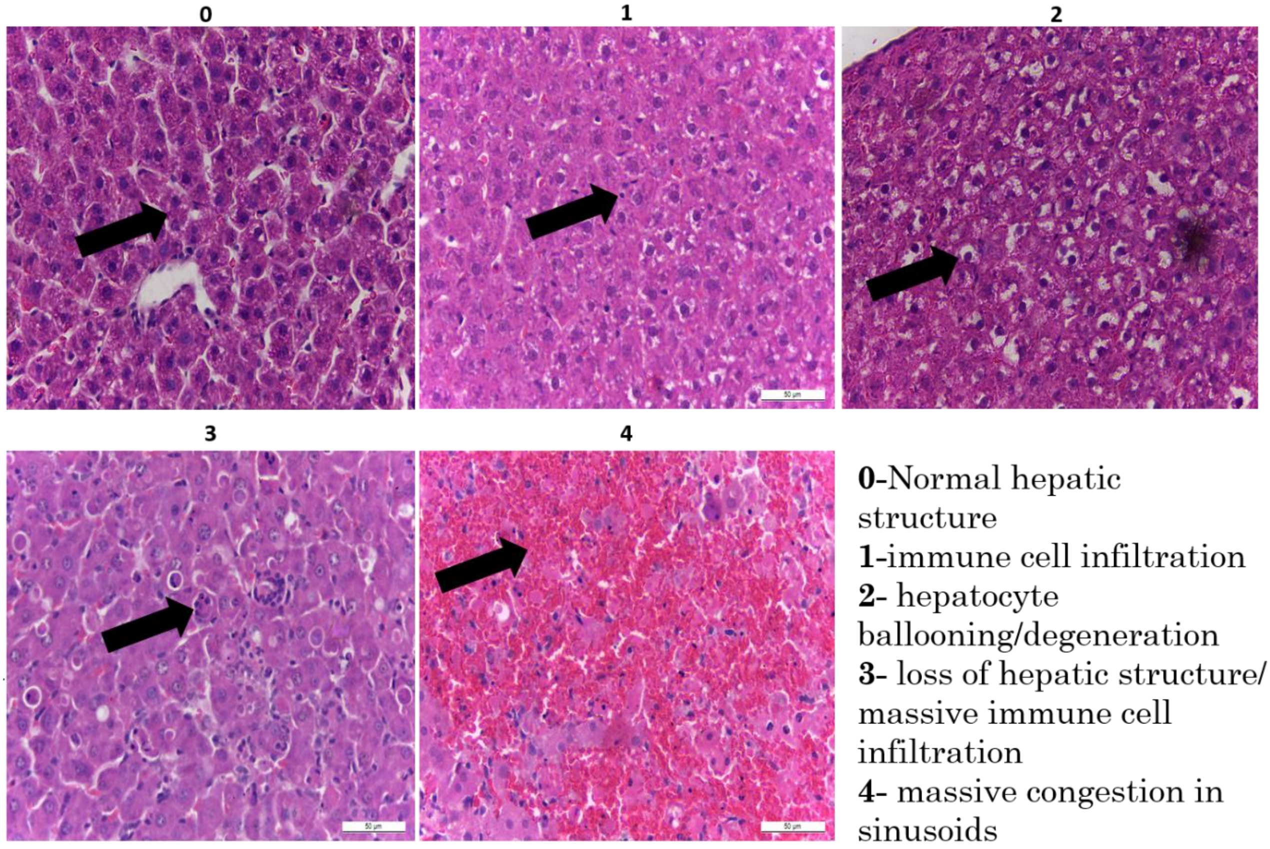

Suppl Fig 1. Representative images of each histopathological score. Bar = 50μM

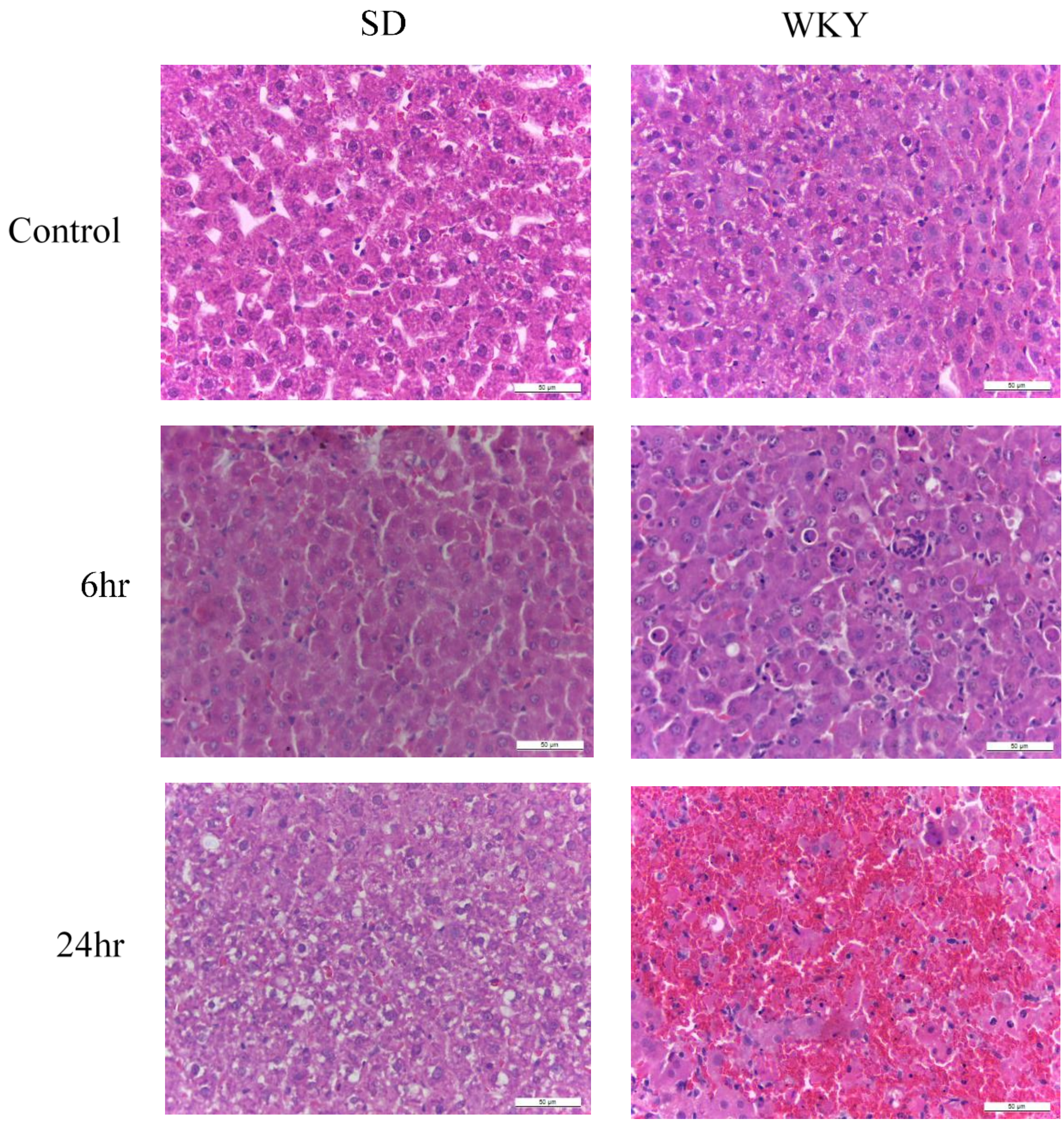

Suppl Fig 2. Histological examination of liver from SD and WKY rats following saline (control) or LPS/GalN administration (6 and 24 hours post administration) (H&E staining x400. Bar = 50μm)).

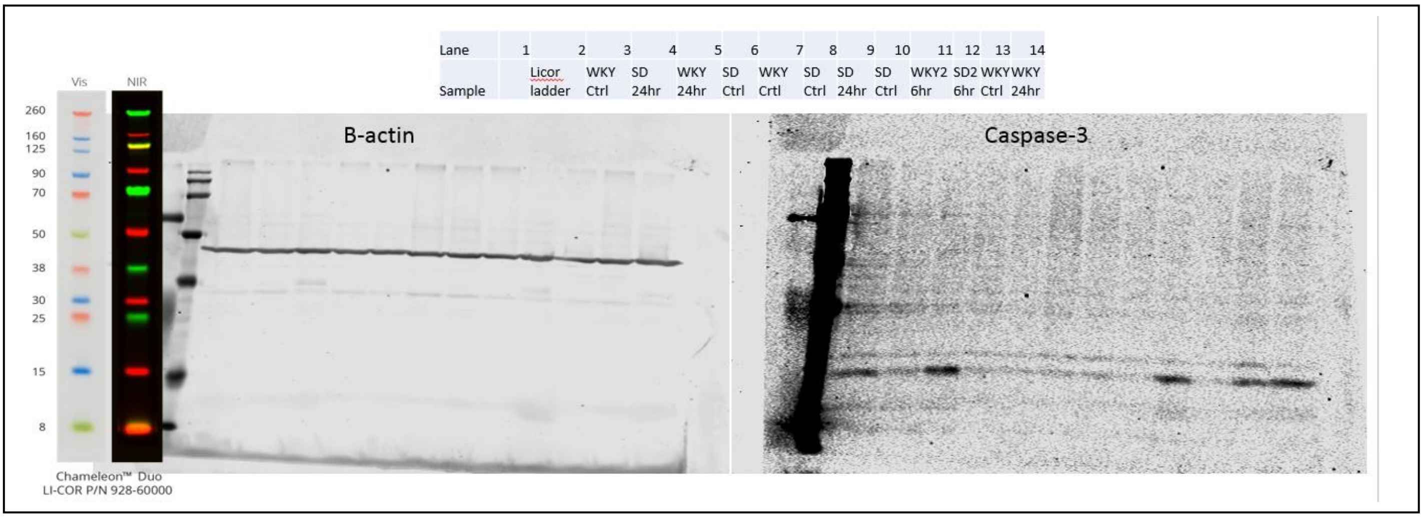

Suppl Fig 3: Representative full western immunoblot image of β-actin (37kDa), Caspase-3 (17 and 19kDa), CB1 *50kDa) and CB2 (34kDa). No effect of WKY or LPS/GalN on expression on expression of 19kDa caspase protein but significant effects on 17kDa expression.
